# Growth-promoting *Sphingomonas paucimobilis* ZJSH1 associated with *Dendrobium officinale* through phytohormone production and nitrogen fixation

**DOI:** 10.1111/1751-7915.12148

**Published:** 2014-08-20

**Authors:** Suijuan Yang, Xinghai Zhang, Zhaoyun Cao, Kaipeng Zhao, Sai Wang, Mingxue Chen, Xiufang Hu

**Affiliations:** 1 College of Life Science, Zhejiang Sci-Tech UniversityRoad 2, Hangzhou, China; 2 Department of Applied Engineering, Zhejiang Economic and Trade PolytechnicXuelin Street 280, Xiasha, Hangzhou, China; 3 China National Rice Research Institute359 Tiyuchang Road, Hangzhou, 310006, China

## Abstract

Growth-promoting *Sphingomonas paucimobilis* ZJSH1, associated with *Dendrobium officinale*, a traditional Chinese medicinal plant, was characterized. At 90 days post-inoculation, strain ZJSH1 significantly promoted the growth of *D. officinale* seedlings, with increases of stems by 8.6% and fresh weight by 7.5%. Interestingly, the polysaccharide content extracted from the inoculated seedlings was 0.6% higher than that of the control. Similar growth promotion was observed with the transplants inoculated with strain ZJSH1. The mechanism of growth promotion was attributed to a combination of phytohormones and nitrogen fixation. Strain ZJSH1 was found using the Kjeldahl method to have a nitrogen fixation activity of 1.15 mg l^−1^, which was confirmed by sequencing of the *nif*H gene. Using high-performance liquid chromatography-mass spectrometry, strain ZJSH1 was found to produce various phytohormones, including salicylic acid (SA), indole-3-acetic acid (IAA), Zeatin and abscisic acid (ABA). The growth curve showed that strain ZJSH1 grew well in the seedlings, especially in the roots. Accordingly, much higher contents of SA, ABA, IAA and c-ZR were detected in the inoculated seedlings, which may play roles as both phytohormones and ‘Systemic Acquired Resistance’ drivers. Nitrogen fixation and secretion of plant growth regulators (SA, IAA, Zeatin and ABA) endow *S. paucimobilis* ZJSH1 with growth-promoting properties, which provides a potential for application in the commercial growth of *D. officinale*.

## Introduction

*Dendrobium officinale* wall. ex Lindl. is a valuable traditional Chinese medicinal orchid (Anon, 1999). As the major officinal part, the stem of *D. officinale* is rich in active compounds, especially polysaccharides (Chen and Guo, 2000). It has been used as the basis for a tonic in Chinese medicine due to possessing immuno-stimulating, anti-tumor and anti-mutagenic activities (Wang *et al*., 2010). Therefore, the demand for *D. officinale* has constantly been increasing, and has resulted in over-exploitation and depletion of this wild plant resource. However, *D. officinale* is usually distributed as a saxicolous epiphyte specie, found at heights ranging from 100∼3000 m, mainly in mountainous regions in Yunnan, Guangxi, Guizhou, Fujian and Zhejiang provinces of China (Lai *et al*., 2006). It usually requires 3–5 years of growth to reach the state of maturity at which it can be effectively utilized as a drug (Waterman and Bidartondo, 2008). Therefore, the supply of wild plants has fallen far short of demand, and artificial cultivation has become the main source of this plant for commercial medicinal use.

At present, *D. officinale* is widely cultivated in the south of China, with a steadily occurring increase in planting area. Because of the small seeds without endosperm characteristic of this plant (Smresiu and Currah, 1989), the supply of seedlings almost completely depends on the tissue culture rather than on seeds (Zhou *et al*., 2012). It normally takes 1 or 2 years to culture the seedlings, and 3 to 5 years for the plantlets to grow, before harvest. A long growth period is accordingly needed for the collection of *D. officinale* as a raw material for medicinal use, which inevitably induces a high price, of over US$1000 kg^−1^ dried stem (Zheng, 2011). Furthermore, the active components, e.g. polysaccharides, are usually found in lesser amounts in the cultivated materials than in the wild counterpart, which makes the former of lower efficacy as a medicine (Si *et al*., 2013). Therefore, it is important to promote the growth of robust seedlings to help improve the survival of the seedlings, and thus promote the growth of the transplants.

Fungi are reported to play a critical role on the growth of *D. officinale*. First, the germination of *D. officinale* benefits from the nutrition of fungi. The seeds do not germinate well without the symbiosis of fungi (Smresiu and Currah, 1989). Second, fungi are the main source of essential nutrients for the growth of *D. officinale* (Waterman and Bidartondo, 2008), which commonly attaches to cliffs usually with small amounts of available nutrients. The plant root cells are usually penetrated by fungal hyphae, and coil-like pelotons are formed within the cortical cells (Clements, 1988). In addition, some fungi can produce antibiotics and plant growth regulators, such as auxins, gibberellic acids, indoleacetic acid, abscisic acid, zeatin and zeatin riboside (Zhang *et al*., 2013).

To the present time, a wide range of fungi, including 38 genera, 11 families, 10 orders, five classes and three subdivisions, have been isolated from *D. officinale* (Li *et al*., 2012b. These fungi, such as *Mycena*, *Rhizoctonia*, *Epulorhiza*, *Alternaria*, *Cephalosporium*, *Acremonium*, *Fusarium*, *Colletotrichum*, *Chaetomium* and *Xylaria*, were found to be beneficial to the germination or growth of *D. officinale* (Guo *et al*., 2000). Many studies have shown that the role of non-mycorrhizal *Dendrobium* fungi in orchid root colonization can stimulate the growth of the host plant (Hou and Guo, 2009).

However, few studies have been reported on the effects of bacteria on *D. officinale.* This is rather surprising, since plants are reported to be closely associated with prokaryotes. Most genera of alpha, beta and gamma *Proteobacteria* and some members of *Firmicutes*, *Bacteriodetes* and *Acinobacteria* have been isolated from plants, and they have been recognized to have considerable favorable impact on plant growth (Ezra *et al*., 2004; Lee *et al*., 2004). Bacteria are also effective agents for stimulating secondary metabolism (Ali *et al*., 2012) and for improving or producing functional components. With regard to *Orchidaceae*, some bacteria were reported to improve seed germination of *Dendrobium moschatum* (Tsavkelova *et al*., 2007). In our previous work (Yu *et al*., 2013), we detected various bacteria in *D. officinale*, with the dominant bacteria genus being *Burkholderia*. Some of these bacteria commonly have the function of nitrogen fixation and thus may potentially play important roles in *D. officinale*. Moreover, bacteria in *Orchidaceae* are primarily concerned with antagonistic action towards plant diseases. Wang and Mo (2009) isolated seven antagonistic bacteria from some wild orchid varieties from Hainan, China. Tsavkelova and colleagues (2001) obtained four bacteria from *D. moschatum* which were all characterized as pathogens. Bacteria with other functions have rarely been reported in association with orchid plants.

Based on the above studies, in the present study we focus on the function of plant-associated bacteria on the growth of seedlings and transplants of *D. officinale*. In our previous research, we isolated 21 bacterial endophytes from surface-sterilized wild *D. officinale* from Qingyuan in Zhejiang Province (data not shown). Among these 21 endophytes, strain ZJSH1 was found to have the potential of growth-promoting activity in preliminary experiments. Herein, the bacterium ZJSH1 will be phenotypically and genotypically characterized, and its effect and mechanism of growth promotion will be investigated.

## Results

Phenotypic and Genotypic characterization of strain ZJSH1 as *Sphingomonas paucimobilis.*

Strain ZJSH1 was phenotypically and genotypically identified. Cultured on nutrient agar (NA) at 28°C for 24 h, the colonies were yellow and smooth edged (Fig. S1B). The cells were aerobic Gram-negative short rods without capsules or spores, and motile with one polar flagellum (Fig. S1C). Tests for oxidase, urease, catalase and for utilization of citrate, fructose and mannose were positive (Table S1). The other enzyme tests, such as for proteinase, lecithinase and amylase, were negative. It is apparent that strain ZJSH1 has phenotypic characteristics in common with *S. paucimobilis* ATCC 31461 (Li and Zhang, 2000).

To further characterize strain ZJSH1, the 16S rRNA gene was amplified and sequenced. Amplification by polymerase chain reaction resulted in a 1450 bp fragment which shared 100% similarity with the 16S rRNA gene of *S. paucimobilis* GIFU2395^T^ (D16144). Phylogenetic analysis revealed that ZJSH1 clustered closely with *S. paucimobilis* and formed a subgroup phylogenetically distinct from other *Sphingomonas* species. The phylogenetic tree (Fig. S2) was constructed from the nucleotide sequence and 18 other homologous sequences, using the neighbor-joining method. The 16S rRNA gene's nucleotide sequence of ZJSH1 was submitted to GenBank with accession number KC017473.

### Growth promotion and polysaccharide accumulation of *D. officinale*

**Fig 1:**
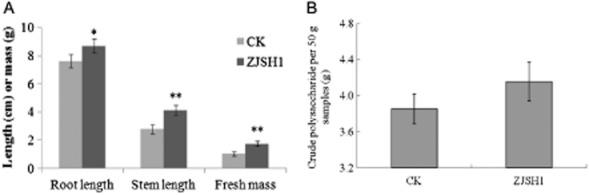
Effects of strain ZJSH1 on growth-promoting of tissue culture seedlings (A) and on polysaccharide accumulation (B).A. Increasing ratio of treatment versus control.B. Polysaccharide content of *D. officinale* seedlings (*P < 0.05; **P < 0.01; CK: control).

According to our preliminary experiments, the optimum inoculum concentration of strain ZJSH1 was 10^4^ CFU ml^−1^ cells. The weights of the fresh seedlings, and the lengths of the roots and the stems, were recorded at 90 days post-inoculation (Fig. 1). As shown in Fig. 1A, the seedlings inoculated with ZJSH1 had higher weights and longer stems and roots than the control seedlings. Only one bacterium, ZJSH1, was then re-isolated from the leaves of the inoculated seedlings of *D. officinale*, demonstrating that the growth promotion was induced by strain ZJSH1.These results indicate that strain ZJSH1 was able to promote the growth of the *D. officinale* seedlings.

**Fig 2:**
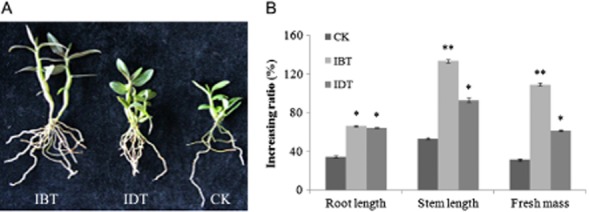
Growth-promoting effect of strain ZJSH1 on the transplants in pot.A. The plants showing stem and root.B. Increasing ratio 10 months post-transplanting versus pre-transplanting (*P < 0.05; **P < 0.01; IBT: inoculation before transplantation; IDT: inoculation during transplantation; CK: control).

The effects of strain ZJSH1 on the growth of the transplants are shown in Fig. 2. According to Fig. 2, it may be seen that at 10 months post-inoculation, the transplants inoculated before transplanting had much higher weights, longer stems and longer roots than both the control group and the group that was inoculated during transplanting. Significantly, the average root length was about 52% greater than that of the control, the average stem length was about 81% greater, and the fresh weight was about 77% greater than that of the control. Inoculation during transplanting also significantly promoted the growth of the seedlings compared with the control. It is evident that inoculation before transplanting had more significant effects on the growth of *D. officinale*, indicating that the earlier inoculation took place, the greater the extent of growth promotion.

The polysaccharides were extracted from the *D. officinale* tissue culture seedlings at 90 days post-inoculation with strain ZJSH1. As shown in Fig. 1B, 4.154 g crude polysaccharide was extracted from 50 g of inoculated *D. officinale,* with a polysaccharide content of 8.3%, while 3.852 g crude polysaccharide was obtained from the control, with a polysaccharide content of 7.7%. However, no polysaccharide was extracted from the fermentation broth of strain ZJSH1 (data not shown), indicating that the polysaccharide originated from the seedlings. It is thus evident that the inoculation of strain ZJSH1 is beneficial for the accumulation of polysaccharides in *D. officinale* seedlings.

### Phytohormone production and nitrogen fixation play a vital role in the growth-promoting properties of *D. officinale*

#### Phytohormones production by strain ZJSH1

The major ingredients in the supernatant of the fermentation broth were further analysed using the LC-MS/MS method described below. As shown in Table 1, 11 types of phytohormones in total were detected in the 2-d culture of strain ZJSH1. Salicylic acid (SA) had the highest concentration of 60.59 ng ml^−1^, and indole-3-acetic acid (IAA) followed with 11.75 ng ml^−1^. Six isomers of zeatin were detected, and their overall total concentration was 5.78 ng·ml^−1^ with c-Z and DZ7G as the major isomers present. In addition, abscisic acid (ABA) and N^6^-isopentenyladenine (Ip and iP7G) were also detected in the fermentation broth. Therefore, strain ZJSH1 appears to produce relatively abundant types of phytohormones, which could be the main reason that it effectively promotes the growth of *D. officinale*.

**Table 1:** Phytohormones produced by endophyte ZJSH1 in fermentation broth

Item	ZJSH1 (ng ml^−1^)
SA (salicylic acid)	60.59 ± 4.56
IAA (indole-3-acetic acid)	11.75 ± 0.90
c-Z (*cis*-zeatin)	2.18 ± 0.11
DZ7G (dihydrozeatin-7-glucoside)	2.11 ± 0.16
Z7G (*trans*-zeatin-7-glucoside)	0.94 ± 0.06
t-Z (*trans*-zeatin)	0.31 ± 0.04
c-ZR (*cis*-zeatin riboside)	0.24 ± 0.01
ZOG (*trans*-zeatin-o-glucoside)	0.02 ± 0.005
ABA (abscisic acid)	1.81 ± 0.06
iP (N^6^-isopentenyladenine)	0.89 ± 0.07
iP7G (N^6^-isopentenyladenine-7-glucoside)	1.21 ± 0.06

#### Phytohormone production in the inoculated plants

**Fig 3:**
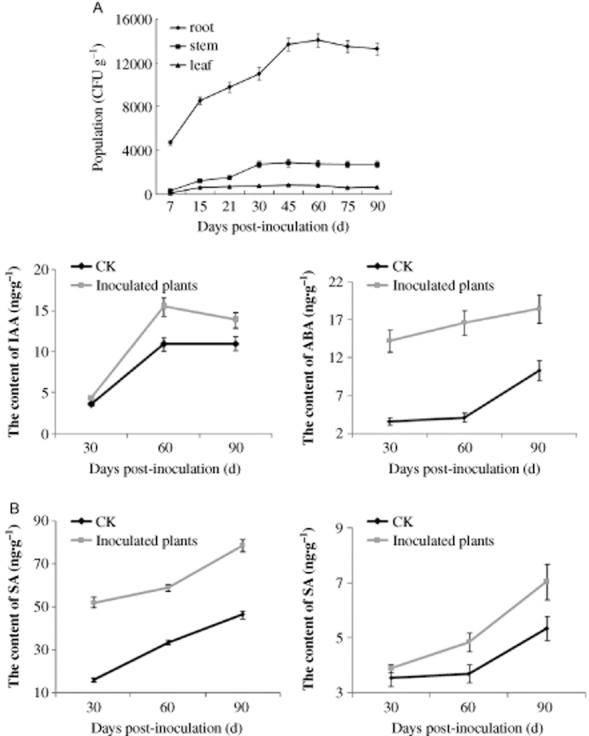
Reproduction dynamics of strain ZJSH1 (A) and their effect on the contents of the four main phytohormones in the culture seedlings (B) (CK: control).

To determine the exact contribution of strain ZJSH1 on the phytohormone production in the inoculated plants, the phytohormone concentration was analysed in the inoculated seedlings. First, the population dynamics of strain ZJSH1 in the seedlings were investigated. As shown in Fig. 3A, the highest population was found within and on the surface of the roots, followed by the stems, and then the leaf samples. During the 90 days growth period of the study, the population of strain ZJSH1 increased continuously during 45–60 days, with a constant population then maintained for the remaining time. The population remained at about 10^4^ CFU g^−1^ in roots, at about 10^3^ CFU g^−1^ in stems and at about 10^2^ CFU g^−1^ in leaves. Therefore, the phytohormone content was accordingly determined at the 30, 60 and 90 days post-inoculation periods (Table S2).

As shown in Fig. 3B, the same phytohormones were detected in plants as in bacterial fermentation broth, and four types of phytohormones were found at levels in the inoculated plants which were higher than in the control groups. Among the four, SA was the phytohormone with the highest concentration, and its level increased over the 90 day period. The SA content was almost twice as high in the inoculated seedlings compared with the controls. Abscisic acid was the second most significant phytohormone, with higher increasing rate detected over the entire 90 days, which was different from that observed for the corresponding content in the supernatant of the fermentation broth. The IAA content showed a rapid increase during the 30–60 day period, with a peak value of 15.50 ng g^−1^, after which it slowly declined over time. The c-ZR content continually increased with higher rate in the later growth term.

#### Nitrogen fixation and phosphate solubilization

Nitrogen fixation and phosphate solubilization are two of the most common mechanisms by which bacteria promote growth in their host. Accordingly, the nitrogen fixation and phosphate solubilization activities were investigated for strain ZJSH1. As shown in Fig. S1A, strain ZJSH1 grew well on nitrogen-free agar plates. In nitrogen-free liquid medium, the turbidity was 1.316 (OD_595_) compared with that of 1.709 in liquid NA, and the total nitrogen content increased to 1.15 mg L^−1^ after culturing for 3 days. The nitrogen fixation-related gene *nif*H was sequenced with accession number KF182367.1, and the resulting sequence shared 99% homology with that of *Stenotrophomonas maltophilia* KNUC170 (GenBank: DQ431165.1). Therefore, the cloning results further confirmed that strain ZJSH1 possessed the activity of nitrogen fixation.

Pikovskaya's agar plate was used to test the phosphate solubilization of ZJSH1. After culturing at 28°C for 3–5 days, no soluble zones were formed, indicating that strain ZJSH1 had no phosphate solubilization activity.

## Discussion

*Dendrobium officinale*, one species of the second largest genus *Dendrobium* in the family *Orchidaceae*, is a valuable traditional Chinese medicinal herb which has been used in China for over 1000 years. Microbes play a vital role in the germination and growth of *D. officinale*, and fungi are commonly the first type of microbe to be considered and studied regarding their function on the plants of *Orchidaceae.* Bacteria, however, are seldom considered in this role. Herein, one particular bacterium, ZJSH1, showed significant growth promotion on both culture seedlings and transplants of *D. officinale*.

The growth-promoting strain ZJSH1 was phenotypically and phylotypically identified as *Sphingomonas paucimobilis*. *Sphingomonas sp.* has a close relationship with the growth of plants. Takeuchi and colleagues (1995) found that *Sphingomonas* sp. could promote the absorption of mineral ions in plants. Sessitsch and colleagues (2004) found that *Sphingomonas* sp. could synthesize siderophores, which promote the absorption of iron ions in plants. Tsavkelova and colleagues (2007) extended beyond individual strains as inoculants and reported an increase in the germination of orchid seeds (*Dendrobium moschatum*) inoculated with *Sphingomonas* sp. and IAA-producing *Mycobacterium* sp. However, *S. paucimobilis* has seldom been studied as a plant-associated bacterium, and to the present time, it has been found to exist in only a few plants, such as potato and *Petunia hybrida* (Garbeva *et al*., 2001; Miyazaki *et al*., 2010). It was recently discovered to be a novel microorganism for biodegrading aromatic compounds, which endows it with potential value in environmental conservation and industrial production (Bending *et al*., 2003). To our knowledge, this is the first time that *S. paucimobilis* has been reported as being a potential plant growth-promoting bacteria (PGPB) in *D. officinale*.

Plant growth promoting bacteria are widely used to improve plant growth. Plant growth-promoting bacteria play vital roles through many ways, including facilitating resource acquisition, by providing plants with resources/nutrients that they lack, such as nitrogen (Iniguez *et al*., 2004) and phosphorus (Kuklinsky-Sobral *et al*., 2004), or by improving the competition for nutrients and niches (Sturz *et al*., 2000). They may also secrete phytohormones (Tsavkelova *et al*., 2006), prevent the proliferation of phytopathogens through inducing systemic resistance or modulate the effects of environmental stress (manifest as heavy metals such as Ca^2+^ and Ni^2+^, salt and drought) or synthesize various antibiotics, lytic enzymes, siderophores, ACC deaminase (Glick, 2012) and detoxification enzymes (Sturz and Christie, 2003).

The growth-promoting mechanism of strain ZJSH1 was determined to be due to a combination of phytohormone excretion and nitrogen fixation. The major phytohormones produced by strain ZJSH1 were SA, IAA, Zeatin and ABA. These phytohormones can be classified into two types according to their function: growth and regulatory hormones (SA, IAA and Zeatin) and stress resistance hormones (SA and ABA). Salicylic acid, a phenolic derivative, is considered to be a potent plant hormone because of its diverse regulatory roles in plants. It has direct involvement in plant growth, thermogenesis, flower induction and uptake of ions (Pérez-Jiménez *et al*., 2013). Enhancement of the levels of chlorophyll and carotenoid pigments and photosynthetic rate, as well as modifying the activity of some of the important enzymes, are other roles assigned to SA, all of which enhance plant growth and yield (Hayat *et al*., 2007). Indole-3-acetic acid, the physiologically most active phytohormone in plants, usually functions as an important signalling molecule in the regulation of plant development, including organogenesis, tropic responses, cellular responses such as cell expansion, division and differentiation and gene regulation (Ryu and Patten, 2008). Zeatin is a member of the plant growth hormone family known as cytokinins, promoting cell division and growth of lateral buds and stimulating cell division to produce bushier plants (Patel *et al*., 2012). These phytohormones may play significant roles on the growth promotion of the seedlings and transplants of *D. officinale*.

Plant growth-promoting bacteria also activate plant defense, resulting in systemic protection against plant pathogens, a phenomenon termed induced systemic resistance (ISR). Some PGPB trigger an SA-dependent signalling pathway by producing nanogram amounts of SA in the rhizosphere (De Meyer and Höfte, 1997). However, the majority of PGPB that activate ISR appear to do so via a SA-independent pathway involving jasmonate and ethylene signals (Pieterse *et al*., 1998). Considerable interest has been generated by the ability of SA to produce protective effects in plants under the actions of biotic stress (Maurhofer *et al*., 1998) and abiotic stresses such as drought, chilling, heavy metal toxicity and osmotic stress (Rivas-San Vicente and Plasencia, 2011). The important role of SA in such protective actions probably determines its ability to induce the expression of genes coding not only for pathogenesis-related protein but also for genes of extensin in *Arabidopsis* plants (Georgios *et al*., 1999). In addition, ABA is understood to mainly contribute to the plant's stress response (Perrig *et al*., 2007). In dry soil, ABA can be transported to the shoots to restrict leaf growth and limit water loss (Dodd, 2005). The stress resistance of these phytohormones is important for the growth of artificial *D. officinale* since the corresponding wild plants favour moist and shady mountainous environments (Lai *et al*., 2006). Such moist and shady environmental conditions are usually not easy to control sufficiently well for artificial plant growth, even under tissue-culturing condition, which places these plants under stress and consequently influences their growth. Therefore, these stress-related phytohormones produced by strain ZJSH1 might protect *D. officinale* from various stresses; however, their effect on the growth needs further confirmation.

The results for the inoculated tissue culture seedlings of the present study showed that strain ZJSH1 became endophytically established mainly in roots from the surface base of seedlings, and then transferred to the stems and leaves, and subsequently the contents of SA, IAA and Zeatin increased. These phytohormones thus may play a significant role on the growth or stress response of the culture seedlings or transplants of *D. officinale*, either singly or in combination through possible synergistic effects. The process through which the phytohormones react on the plants of *D. officinale* requires further detailed study.

*Dendrobium officinale* usually grows on nutrient-poor cliffs on mountains (Lai *et al*., 2006). It grows slowly and does not grow well without the presence of microbes (Waterman and Bidartondo, 2008). These facts could explain the growth-promoting effect of strain ZJSH1. Until now, there have been few reports concerning the study of strains which have the ability of nitrogen fixation in orchid plants, and especially in *Dendrobium*. Only associative cyanobacteria and some strains of *Pseudomonas* and *Bacillus* were isolated from the orchids *Calanthe vestita* var. rubro-oculata and *D. moschatum*, and some of these were assessed as having nitrogen fixation activity (Tsavkelova *et al*., 2001). In the present study, strain ZJSH1 has the activity of nitrogen fixation which would assist nitrogen absorption in *D. officinale*, and the nitrogen fixation-related gene was cloned and the sequence had 99% similarity with the *nif*H gene of *Stenotrophomonas maltophilia* KNUC170 (GenBank: DQ431165.1). The present study is the first in which *Sphingomonas sp.* has been found to have nitrogen fixation activity. The extent to which strain ZJSH1 contributes to the providing of nitrogen for the growth of *D. officinale,* and its distribution in *D. officinale* need to be elucidated through further research.

## Experimental procedures

### Materials

The plant-associated bacterium ZJSH1, isolated from surface-sterilized roots of *D. officinale* by our laboratory and stored as No: M 2010041 in the China Center for Type Culture Collection (CCTCC), was routinely cultured on NA at 28°C. The sterile tissue culture seedlings of *D. officinale* were prepared in our laboratory according to the protocol described by Li and colleagues (2012a).

### Characterization of strain ZJSH1

#### Phenotypic characteristics

Strain ZJSH1 was inoculated and cultured on NA for 24 h at 28°C, and phenotypical analysis was conducted by analyzing its morphology, gram staining and biochemical characteristics. Cell morphology was examined using a light microscope following Gram staining. Microscopic cell morphology and flagella of strain ZJSH1 were observed by transmission electron microscopy (JEM 1200 EX) after negative staining with 1% uranyl acetate. Physiological tests, including aerobic growth, Methyl Red and Voges–Proskauer reactions, production of acid from carbohydrates, utilization of organic acids, NaCl tolerance, gelatin liquefaction and enzyme activities, were performed by the methods of Claus and Berkeley (1986).

#### Genotypic characterization and phylogenetic analysis

For genotypic characterization, genomic DNA extraction, amplification and sequencing of 16S rRNA and *nif*H genes were performed as previously described (Hu *et al*., 2009). The sequencing was carried out by Shanghai Invitrogen Biotechnology Company, Limited (Shanghai, China). The nucleotide sequences of 16S rRNA and *nif*H genes were aligned with representatives from similar genera deposited in the GenBank, EMBL and DDBJ databases using the program MEGA version 4.1. The phylogenetic trees based on the 16S rRNA gene were constructed by three methods: the neighbor-joining (mega version 3.1), maximum parsimony and maximum likelihood (Phylip version 3.65) (Saitou and Nei, 1987) methods. Bootstrap re-sampling analysis was performed to estimate the confidence of the tree topologies.

#### Growth-promoting effect of strain ZJSH1 on *D. officinale*

To evaluate the growth-promoting effect on *D. officinale*, strain ZJSH1 was inoculated on the tissue culture seedlings and transplants of *D. officinale*.

The inoculum was cultured by transferring strain ZJSH1 to a 250 ml Erlenmeyer flask containing 50 ml nutrient broth, followed by incubation at 28°C on a shaker (220 r.p.m.) for 2 days. After the incubation period, the culture was centrifuged and diluted into 10^4^ CFU ml^−1^ using double-distilled (dd) H_2_O. A 50-μL bacterial suspension was inoculated on the base of each of the tissue culture seedlings of *D. officinale*, which were 45 days old, while the control was treated only with 50 μL dd H_2_O instead. The tissue culture seedlings were grown in a greenhouse under conditions of 25°C constant temperature with a 12 h sustained photoperiod. After 90 days of inoculation, the growth (height and fresh weight) and quality (content of polysaccharides) of the seedlings were determined. Fifteen replicates were used for each treatment, and the experiment was repeated in triplicate.

Some of the tissue culture seedlings above were used for transplanting. Before transplanting, all the seedlings were rinsed with sterilized tap water to remove the medium, and air-dried for 12 h indoors, which helps to remove some water and thus raise survival rate. The seedlings previously inoculated with strain ZJSH1 were used as the inoculation treatment for potted culturing before transplanting. The seedlings without inoculation of ZJSH1 were divided into two portions before being transplanted. The first portion was used for seedling inoculation through soaking in bacterial suspension (10^4^ CFU ml^−1^) for 10 min, prepared as inoculation treatment during transplanting. The second portion was used for the non-inoculated seedlings as a control. Each treatment was replicated in triplicate, and each replicate consisted of 10 pots. The cracked bark of pine trees was used as the cultivation medium for the potted culture experiments. All the plants were sprayed with water every day. The effect of the bacteria on the pot-cultured *D. officinale* was investigated by measuring the roots, stems and fresh weights of all groups.

#### Polysaccharide content

As the major active ingredient, polysaccharide content is usually determined to evaluate the quality of *D. officinale* seedlings. The water extraction and alcohol precipitation method (Luo *et al*., 2009) was adopted to extract the polysaccharides of the inoculated and non-inoculated 90-day-old tissue culture seedlings of *D. officinale*. Prior to extraction, the stems were thoroughly washed using tap water, dried at 60°C for 5 h and ground with liquid nitrogen. After grinding, 50 g of the powder were treated with 80% ethanol for 1 h, and then filtered. After filtering, the residue was further extracted with 1000 ml distilled water. All extracts were combined and then concentrated to 100 ml under low pressure. The extracts were precipitated with 400 ml of 95% ethanol, and the mixtures were kept overnight at 0°C. Finally, the precipitate was washed successively with ethanol and acetone (each for five times), and recovered by freeze-drying, which produced a crude polysaccharide sample.

### Growth promotion mechanism analysis

#### Phytohormones produced by ZJSH1 in medium and in plants

Phytohormones are usually involved in promoting plant growth. The contents of phytohormones in the fermentation broth and in the inoculated seedlings were analysed. To detect the production of phytohormones, strain ZJSH1 was inoculated into a flask with nutrient broth and then cultured at 28°C with shaking at 220 r.p.m. for 2 days. After centrifugation to remove the cells, the supernatant was decanted and used for analysis of phytohormones.

To detect the contents of phytohormones in the inoculated seedlings, a 50 mg sample of stem was taken from the inoculated plants, and frozen with liquid nitrogen. The plant tissues were then ground into powder, to which 50 μl of the internal standard working solution were added. The microscale extraction and centrifugation process was performed using the method described by Pan and colleagues (2010). The supernatant was used for phytohormone analysis by LC-MS/MS.

##### HPLC conditions

High-performance liquid chromatography analysis was performed using a Surveyor HPLC System (Thermo Scientific, San Jose, CA). Chromatographic separation was carried out using a 100 mm × 2.1 mm inner diameter LC column (ZORBAX Extend-C18, 1.8 μm particle size, Agilent Technologies, Lawrence, USA,). The binary solvent system consisted of water with 5 mmol L^−1^ formic acid (A) and methanol (B) as mobile phase. The column temperature was maintained at 40°C, and the flow rate was 150 μl min^-1^. The injection volume was 2 μl.

##### Mass spectrometry analysis

Mass spectrometry (MS) analysis was carried out on a TSQ Quantum Access MAX triple stage quadrupole mass spectrometer with a heated electrospray ionization (HESI) probe (Thermo Scientific, San Jose, CA). The MS conditions were as follows: ion source polarity: negative or positive; spray voltage: −2200 V or 2800 V; vaporizer temperature: 300°C; skimmer offset: 3 V; sheath gas pressure (N_2_): 30 (arbitrary units); auxiliary gas pressure (N_2_): 5 (arbitrary units); collision pressure: 1.2 m Torr; ion transfer tube temperature: 300°C; scan type: selective reaction monitoring.

In order to elucidate the exact contribution of endophyte on the effect of phytohormones in plants, the growth dynamics of inoculated bacteria were investigated. Therefore, endophytic bacteria were simultaneously isolated from the roots, stems and leaves of the inoculated tissue culture seedlings of *D. officinale* every other week. After washing the seedlings using sterile tap water, 1 g each of root, stem and leaf samples was pulverized and diluted in dd H_2_O, and spread on the NA medium. The resulting bacterial populations were calculated using the plate counting method.

#### Phosphate solubilization

The phosphate-solubilizing activity of strain ZJSH1 was determined by measuring the size of the transparent zone formed by solubilization of insoluble phosphate on Pikovskaya's agar plates [1% C_6_H_12_O_6_, 0.5% Ca_3_(PO_4_)_2_, 0.05% (NH_4_)_2_SO_4_, 0.02% NaCl, 0.01% MgSO_4_.7H_2_O, 0.02% KCl, 0.05% yeast extract, 0.0002% MnSO_4_.H_2_O, 0.0002% FeSO_4_.7H_2_O, pH 7.2]. The zone of clearance around the colony was observed at 3–5 days post-inoculation.

#### Nitrogen fixation

To estimate the nitrogen fixation ability, strain ZJSH1 was qualitatively and quantitatively analysed. For the qualitative test, strain ZJSH1 was both inoculated and cultured for 3–5 days in a liquid nitrogen-free medium on the same plate. The nitrogen fixation ability was evaluated by the growth, the colony diameter sizes on the plate and OD_595_nm in liquid medium. For the quantitative test, strain ZJSH1 was incubated in a flask with nitrogen-free liquid medium and then cultured at 28°C with shaking at 220 r.p.m. for 2 days. The nitrogen fixation ability was quantitatively evaluated by the increase of total nitrogen which was measured using the Micro-Kjeldahl method (Allen, 1931). The nitrogen-free medium consisted of 1% mannitol, 0.02% KH_2_PO_4_, 0.02% MgSO_4_·7H_2_O, 0.02% NaCl, 0.5% CaCO_3_, 0.01% CaSO_4_·2H_2_O, pH 6.8–7.0. To confirm the nitrogen fixation ability, the gene encoding nitrogenase was further cloned using the primers PolF (5′-TGCGAYCCSAARGCBGACTC-3′) and PolR (5′-ATSGCCATCATYTCRCCGGA-3′) described by Doreen and colleagues (2012).

### Statistical analysis

The experimental data were statistically analysed by a *t*-test using the program dps 7.05 (Tang and Feng, 2007). Values are given as means ± SD for each sample, and differences were considered to be significant at the *P* < 0.05 level.

## Conclusions

The growth-promoting bacterium ZJSH1 of *D. officinale*, with the ability to fix nitrogen and to excrete plant growth regulators such as SA, IAA, Zeatin and ABA, was investigated in this study and identified as *S. paucimobilis*. This growth-promoting strain may provide a more environmentally safe and efficient alternative to solving the problems existing in the artificial planting and cultivation of *D. officinale*.

## Supporting information

**Fig. S1.** The growth of strain ZJSH1 on nitrogen-free agar plate (A) and morphologies of colonies (B) and cells (C) of strain ZJSH1. **Fig. S2.** Phylogenetic tree showing the relationship between strain ZJSH1 and its closely related strains. The tree was based on alignment of 16S rRNA gene sequences and was constructed using the Neighbor Joining method (mega version 4.1). The sequence of *Escherichia coli* was used as an out-group. Stability of the tree was assessed by 1000 bootstrap replications with Felsenstein confidence limits. **Table S1.** Phenotypic characteristics of endophyte ZJSH1 compared with *Sphingomonas paucimobilis* **ATCC 31461**. **Table S2.** Auxin detected in the seedlings inoculated with or without endophyte ZJSH1.
