# Supplementary material for: Growth-promoting Sphingomonas paucimobilis ZJSH1 associated with Dendrobium officinale through phytohormone production and nitrogen fixation
Source: Microb Biotechnol. 2014 Aug 20;7(6):611–20. doi: 10.1111/1751-7915.12148 (PMC4265079; doi:10.1111/1751-7915.12148)
Supplement: Supplementary file 1 [file mbt20007-0611-sd1.doc]

Supplementary Material

**Growth-Promoting *Sphingomonas paucimobilis* ZJSH1 Associated with *Dendrobium officinale* through Phytohormone Production and Nitrogen Fixation**

Kaipeng Zhao^1^, Suijuan Yang^1^, Xinghai Zhang^2^, Zhaoyun Cao^3^, Sai Wang and Xiufang Hu^1^*

^1^ College of Life Science, Zhejiang Sci-Tech University, Road 2, Hangzhou, China. ^2^ Department of Applied Engineering, Zhejiang Economic and Trade Polytechnic, Xuelin Street 280, Xiasha, Hangzhou, China. ^3^ China National Rice Research Institute, 359 Tiyuchang Road, 310006, Hangzhou, China.

**Author for correspondence:**

Professor Dr. Xiu-fang Hu E-mail: huxiuf@zstu.edu.cn

Mailing address: College of Life Science, Zhejiang Sci-Tech University, Road 2, Xiasha, Hangzhou 310018, P. R. China

Phone: 86-571-86843195 Fax: 86-571-86843196

Table S1. Phenotypic characteristics of endophyte ZJSH1 compared with *Sphingomonas paucimobilis* **ATCC 31461** (Li and Zhang, 2000)

| **Item** | **ZJSH1** | **ATCC 31461** | **Item** | **ZJSH1** | **ATCC 31461** |
| --- | --- | --- | --- | --- | --- |
| Shape | Short rod | Short rod | Cellobiose | － | － |
| Motility | + | + | L-Arabinose | － | － |
| Aerobic | + | / | Nitrate reduction | － | － |
| Gram stain | － | － | Malonate | － | － |
| Spore | － | － | Citrate | + | + |
| Capsule | － | － | V-P test | － | － |
| Growth temprerature | 10°C ~40°C, best at  28°C ~37°C | 10°C~41°C | H_2_S production | － | － |
| Growth at NaCl | 3%~8% | / | Indol | － | － |
| Growth pH | 6.0~9.0 | / | Oxidase | + | + |
| α-Glucose | － | － | Catalase | + | + |
| Saccharose | － | － | Gelatinase | － | － |
| Maltose | － | － | Urease | + | + |
| Fructose | + | + | Proteinase | － | / |
| Mannose | + | + | Lecithinase | － | / |
| Rhamnose | － | － | Amylase | － | / |

Note: “+”represents positive reaction, “－”represents negative reaction

Table S2 Auxin detected in the seedlings inoculated with or without endophyte ZJSH1

| **Group** | **IAA**  **(ng/g)** | **IAA-Asp**  **(ng/g)** | **SA**  **(ng/g)** | **ABA**  **(ng/g)** | **c-ZR**  **(ng/g)** | **iPR**  **(ng/g)** |
| --- | --- | --- | --- | --- | --- | --- |
| **30d** | 4.39±0.26^d^ | 7092.36  ±261.73^a^ | 52.13±0.96^b^ | 14.26±1.41^b^ | 3.88±0.31^c^ | 0.94±0.07^d^ |
| **30d-CK** | 3.57±0.15^d^ | 6758.76  ±99.50^b^ | 16.12±2.51^c^ | 3.62±0.52^d^ | 3.55±0.15^c^ | 1.38±0.12^b^ |
| **60d** | 15.50±1.75^a^ | 6583.42  ±448.01^b^ | 59.02±4.25^b^ | 16.67±2.42^b^ | 4.84±0.33^b^ | 0.77±0.07^e^ |
| **60d- CK** | 10.91±0.40^c^ | 4434.42  ±434.51^c^ | 33.49±1.19^c^ | 4.11±0.61^d^ | 3.69±0.33^c^ | 1.17±0.24^c^ |
| **90d** | 13.87±0.26^b^ | 4513.73  ±75.15^c^ | 78.57±1.57^a^ | 18.50±3.09^a^ | 7.05±0.45^a^ | 1.38±0.15^b^ |
| **90d-CK** | 10.96±0.38^c^ | 3742.74  ±392.94^d^ | 46.36±2.75^b^ | 10.37±1.88^c^ | 5.35±0.65^b^ | 1.48±0.29^a^ |

Fig.S1


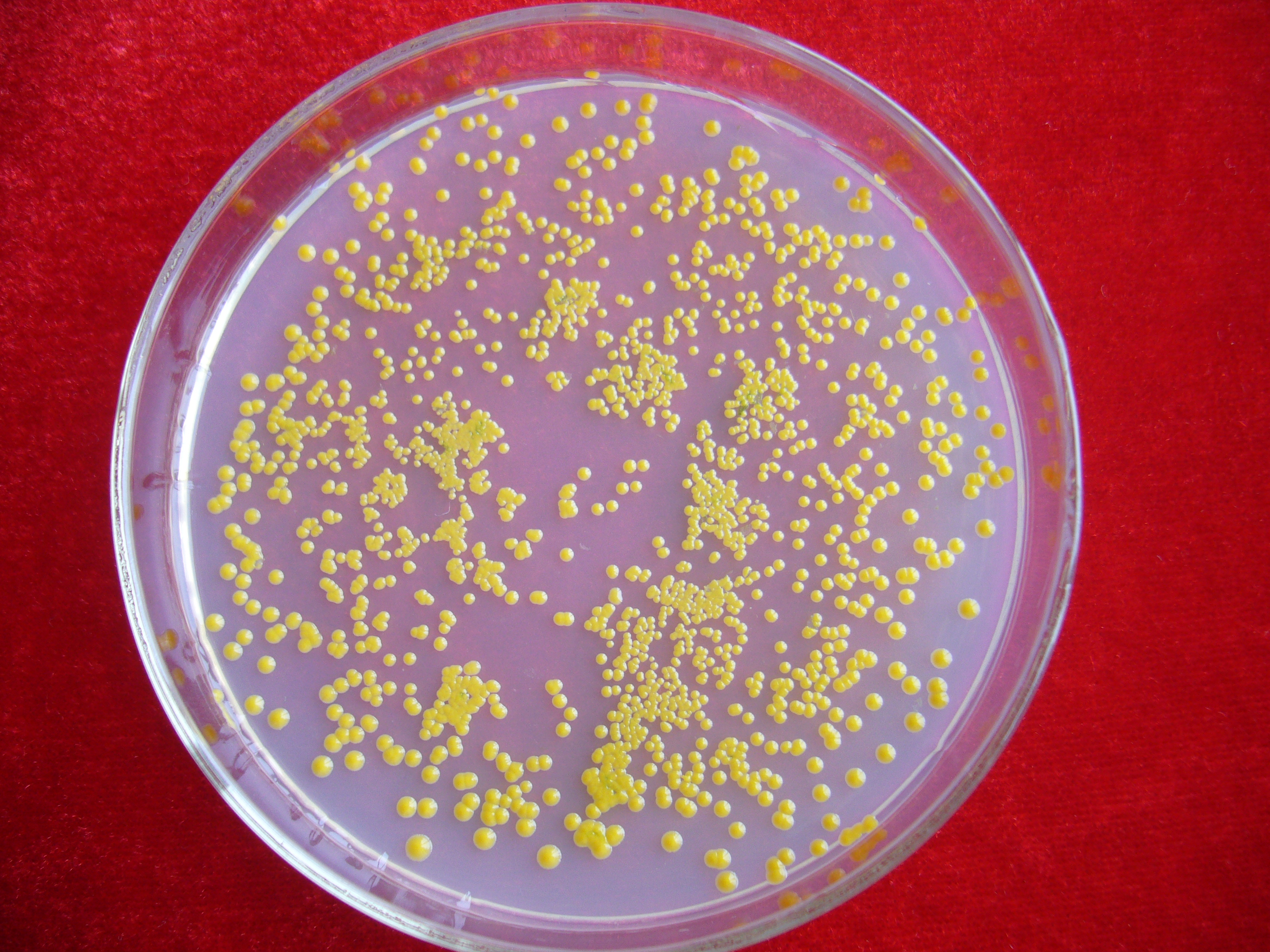

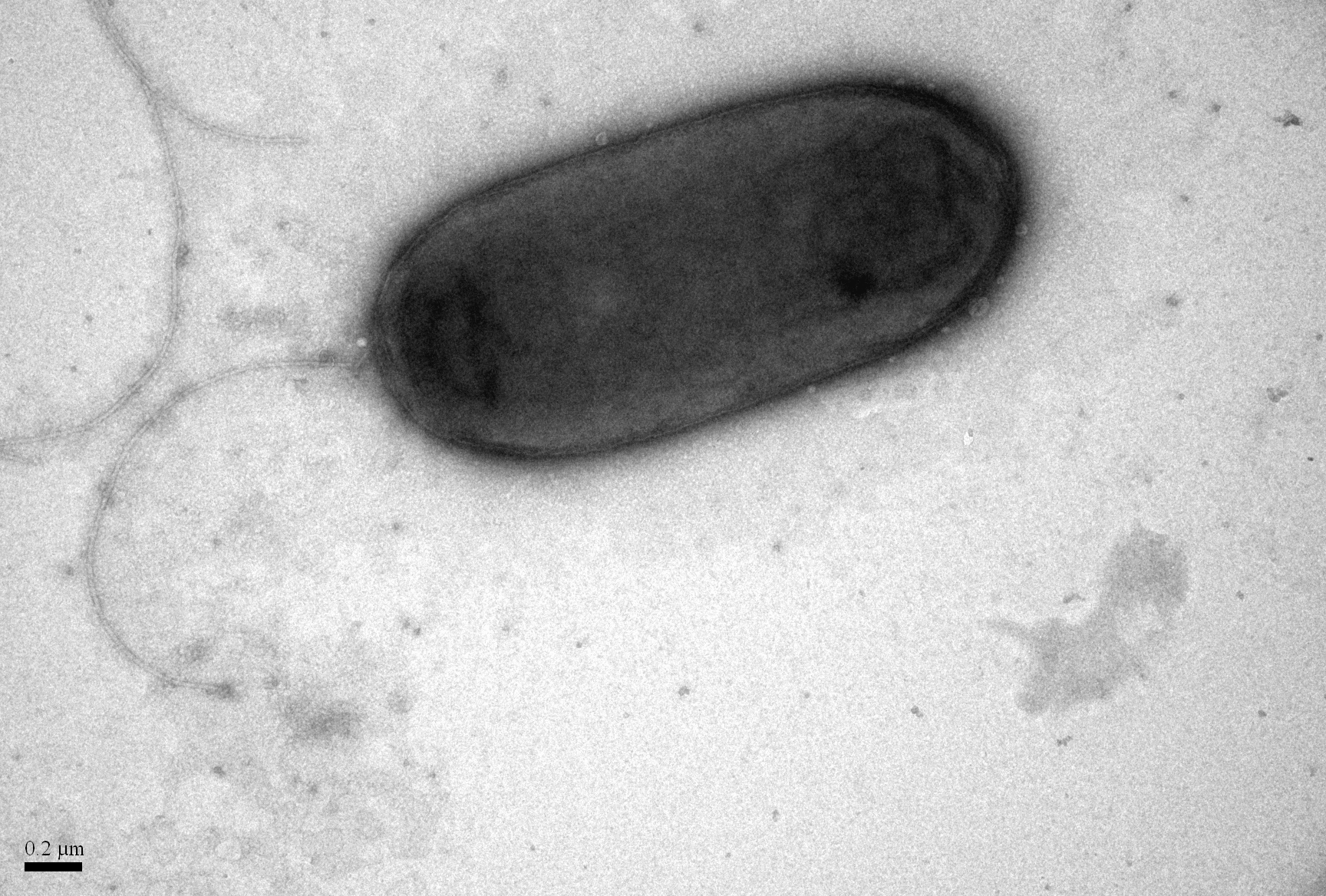

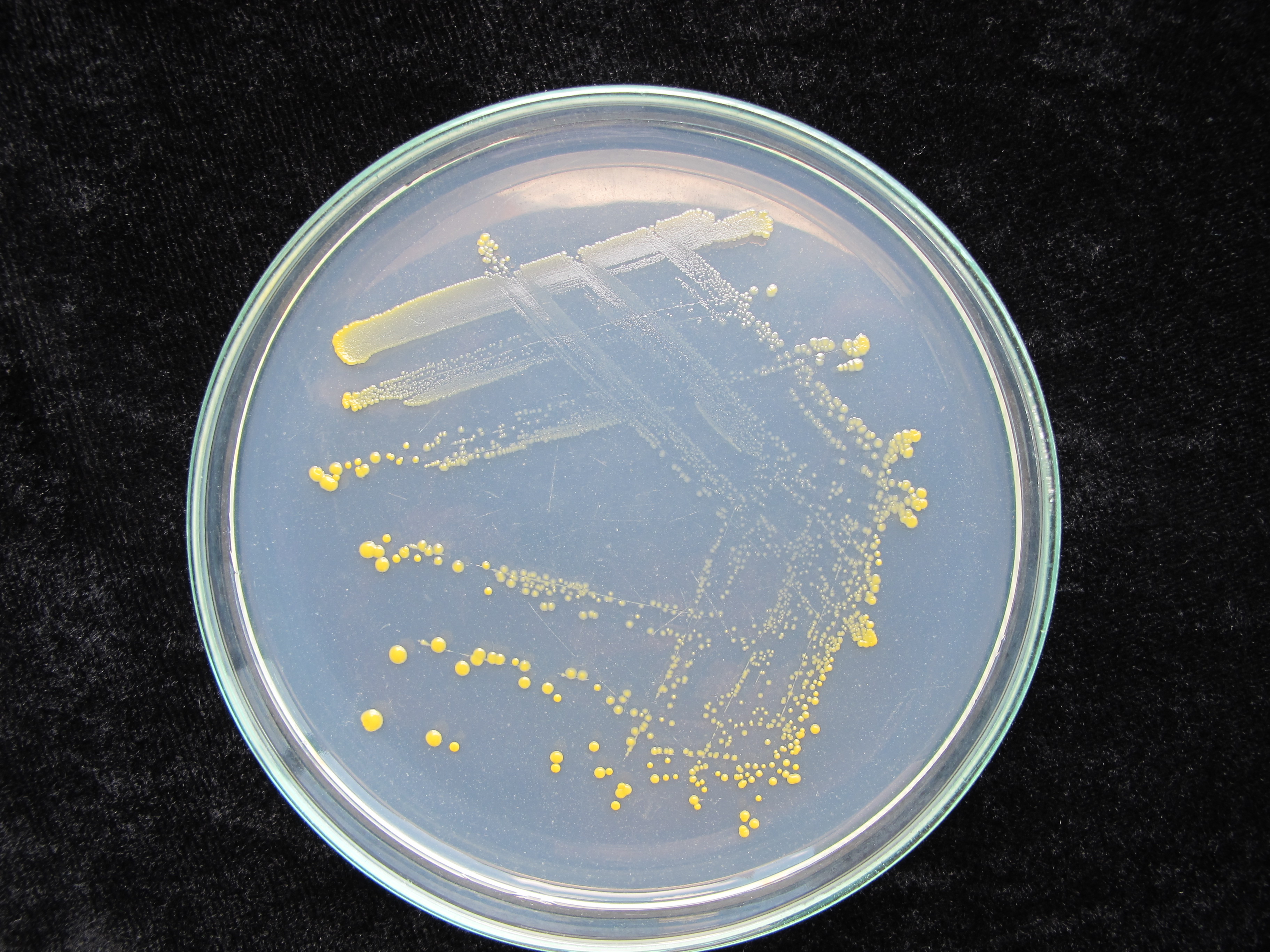


**A**

**B**

**C**

Fig.S2

*Sphingomonas insulae* DS-28^T^(EF363714)

*Sphingomonas* sp*.* JSS-7^T^(AF131295)

*Sphingomonas aestuarii* K4^T^(EF660755)

*Sphingomonas* sp*.* JSS-26^T^(AF131296)

*Sphingomonas desiccabilis* CP1D^T^(AJ871435)

*Sphingomonas* sp*.* ODN7^T^(FJ194436)

*Sphingomonas dokdonensis* DS-4^T^(DQ178975)

*Sphingomonas japonica* KC7^T^(AB428568)

*Sphingomonas* sp*.* 15497^T^(AB033947)

**ZJSH1 (KC017473)**

*Sphingomonas paucimobilis* GIFU2395(D16144)

*Sphingomonas abaci* C42^T^(AJ575817)

*Sphingomonas astaxanthinifaciens*^T^(AB277583)

*Novosphingobium capsulatum*^T^(D16147)

*Sphingomonas adhaesiva*^T^(D13722)

*Sphingomonas* sp*.*^T^(Z73631)

S*phingopyxis macrogoltabida*^T^(D13723)

*Escherichia coli* ATCC 11775^T^(X80725)

97

100

100

68

94

62

62

85

63

99

27

34

20

30

37

0.02
